# Pre-Surgical Integration of fMRI and DTI of the Sensorimotor System in Transcortical Resection of a High-Grade Insular Astrocytoma

**DOI:** 10.3389/fnint.2016.00015

**Published:** 2016-03-11

**Authors:** Chelsea L. Ekstrand, Marla J. S. Mickleborough, Daryl R. Fourney, Layla A. Gould, Eric J. Lorentz, Tasha Ellchuk, Ron W. Borowsky

**Affiliations:** ^1^Department of Psychology, University of SaskatchewanSaskatoon, SK, Canada; ^2^Department of Medical Imaging, Royal University HospitalSaskatoon, SK, Canada; ^3^Department of Surgery, Division of Neurosurgery, Royal University HospitalSaskatoon, SK, Canada

**Keywords:** insular astrocytoma, fMRI, DTI, motor and sensory localization, pre-surgical planning

## Abstract

Herein we report on a patient with a WHO Grade III astrocytoma in the right insular region in close proximity to the internal capsule who underwent a right frontotemporal craniotomy. Total gross resection of insular gliomas remains surgically challenging based on the possibility of damage to the corticospinal tracts. However, maximizing the extent of resection has been shown to decrease future adverse outcomes. Thus, the goal of such surgeries should focus on maximizing extent of resection while minimizing possible adverse outcomes. In this case, pre-surgical planning included integration of functional magnetic resonance imaging (fMRI) and diffusion tensor imaging (DTI), to localize motor and sensory pathways. Novel fMRI tasks were individually developed for the patient to maximize both somatosensory and motor activation simultaneously in areas in close proximity to the tumor. Information obtained was used to optimize resection trajectory and extent, facilitating gross total resection of the astrocytoma. Across all three motor-sensory tasks administered, fMRI revealed an area of interest just superior and lateral to the astrocytoma. Further, DTI analyses showed displacement of the corona radiata around the superior dorsal surface of the astrocytoma, extending in the direction of the activation found using fMRI. Taking into account these results, a transcortical superior temporal gyrus surgical approach was chosen in order to avoid the area of interest identified by fMRI and DTI. Total gross resection was achieved and minor post-surgical motor and sensory deficits were temporary. This case highlights the utility of comprehensive pre-surgical planning, including fMRI and DTI, to maximize surgical outcomes on a case-by-case basis.

## Introduction

We report on a 44-year-old female with a 30-month history of progressive headache who, for about 4 months prior to diagnosis, experienced simple partial seizures. The seizures manifest as numbness affecting the right arm and leg several times daily. During ictus, she also reported mild word-finding difficulty and trouble organizing her thoughts. Her neurological examination was normal. Diagnostic magnetic resonance imaging (MRI) revealed a 7 × 4 cm, heterogeneous, non-enhancing intra-axial mass in the right insular region. Perfusion imaging showed decreased cerebral blood volume, while magnetic resonance spectroscopy (MRS) demonstrated a mildly depressed N-acetylaspartate (NAA) and an elevated choline, with no appreciable lactate doublet. Choline mapping revealed a “hot spot” which was targeted for biopsy. Interestingly, while the diagnostic MRI would suggest a low-intermediate grade astrocytoma, the stereotactic biopsy, guided by the choline map confirmed a WHO Grade III astrocytoma.

## Background

Resection of insular gliomas remains surgically challenging based on their close proximity to the internal capsule, frequently leading to impairment of the corticospinal tract (Sanai et al., 2010). However, it is also well known that the extent of tumor resection is positively related with lower rates of recurrence as well as improved long-term tumor control (Berger et al., 1994; Keles et al., 2001; Smith et al., 2008). For example, 2-year survival rate of patients with high-grade insular gliomas increases from 75% to 91% as the extent of resection increases from less than 90% to greater than 90% (Sanai et al., 2010). Thus, maximal resection must be balanced with surgically related adverse outcomes. The magnitude and safety of the resection can be optimized through pre-surgical planning techniques to provide the surgeon with comprehensive information to minimize surgical risks and improve post-surgical outcomes.

Functional MRI (fMRI) and diffusion tensor imaging (DTI) are becoming increasingly common in pre-surgical planning, as they allow for noninvasive identification of important functional areas involved in predefined cognitive tasks and for tracking white matter fiber bundles, respectively (Staempfli et al., 2008). In this case study, fMRI and DTI were used to inform the surgical approach in the gross-resection of a high-grade insular astrocytoma. As such, pre-surgical planning integrating fMRI and DTI may help to identify, and subsequently allow surgeons to avoid, areas of important functional and anatomical redistribution.

Prior to surgical resection, fMRI localization of motor and sensory processing as well as DTI reconstruction of the motor and sensory tracts was used to inform resection trajectory and allow for maximal tumor resection while avoiding important white matter tracts. Functional data pertaining to language processing was also acquired, which confirmed left language dominance and, thus, language processing was contra-lateral to the side of resection. This research was approved by the University of Saskatchewan Biomedical Research Ethics Board and the patient gave written informed consent in accordance with the Declaration of Helsinki.

## Materials and Methods

### fMRI Protocol

All imaging was conducted on a 3 Tesla Siemens Skyra scanner. Whole-brain anatomical scans were acquired using a high resolution magnetization prepared rapid acquisition gradient echo (MPRAGE) sequence consisting of 192 T1-weighted echo-planar images (EPI) slices of 1 mm thickness (no gap) with an in-plane resolution of 1 × 1 mm (field of view 256; TR = 1900 ms; TE = 2.08 ms). For each of the functional tasks, T2*-weighted single shot gradient-echo EPI scans were obtained via an interleaved ascending EPI sequence, consisting of 55 volumes of 25 axial slices of 4 mm thickness (1 mm gap) with an in-plane resolution of 2.65 × 2.65 mm (field of view = 250) using a flip angle of 90°. The top two coil sets (16 channels) of a 20-channel Siemens head-coil were used, with the bottom set for neck imaging (4 channels) turned off. To minimize motion artifact, we used a sparse-sampling (gap paradigm) fMRI method where the motor task occurred during a gap in image acquisition (TR = 3300 ms, with a 1650 ms gap of no image acquisition; TE = 30 ms; Flip Angle = 90°; Borowsky et al., 2007, 2013). The acquisition matrix was 94 × 94.

### DTI Protocol

DTI imaging was conducted on a 3 Tesla Siemens Skyra MRI scanner in the same session as the T1 and fMRI data, with images acquired using a dual spin-echo single-shot echo-planar imaging sequence. Twenty non-collinear directions of diffusion-sensitizing gradients were acquired, diffusion sensitivity *b* = 1000 s/mm^2^, TR = 3700 ms, TE = 95 ms. Twenty-five, 4 mm thick axial slices were obtained. The voxel resolution was 1.72 × 1.72 × 4 mm, with a field of view of 220 mm and an acquisition matrix of 128 × 128.

### Behavioral Tasks

In order to optimally activate regions proximal to the tumor, we developed a novel set of three experimental sensorimotor tasks: rubbing arms against the scanner; rubbing hands against the scanner; and licking of lips. Both motor and sensory activation occurred simultaneously in the relevant/respective anatomic regions. These tasks were chosen based on the location of the tumor and its close proximity to the sensorimotor cortices associated with the hands and upper limbs. Also, based on the planned frontotemporal approach, these tasks would elicit the most relevant activation in close proximity to the tumor and therefore be most useful in pre-surgical planning. The patient performed five blocks with five trials per block for each sensory and motor task and the trials were synchronized with the onset of each acquisition volume. During these blocks, the word “touch” was presented on the screen for 16.5 s (i.e., 5 × TR = 3300 ms) during which the participant was required to perform the relevant motor task. This was followed by 16.5 s of rest. The patient also performed four language tasks to localize language areas, to ensure that they were not near the resection zone: exception word reading (words that cannot be decoded phonetically in order to be read correctly; e.g., “yacht”); pseudohomophone reading (non-words that when decoded phonetically sound like real words; e.g., “yawt”); picture naming; and answering semantic questions (e.g., “what do you shave with?”). The patient reported no difficulties or discomfort performing these tasks throughout the duration of the trials.

### fMRI Analyses

All preprocessing and statistical analyses for functional images were performed using Brain Voyager QX Version 2.6.1^1^. Functional images were preprocessed and corrected for slice scan time acquisition (cubic spline interpolation), 3D motion correction (trilinear/sinc interpolation), and temporal filtering with a high-pass (GLM-Fourier) filter to remove frequencies less than two cycles/time course. The functional data were assessed for head motion and/or magnet artifacts, in which there was less than 1 mm of motion in any of the three translational or three rotational parameters for each of the tasks. The first five image volumes used to achieve steady state of image contrast were discarded prior to analysis. A fixed effects model was used, whereby the hemodynamic response function was smoothed using a convolution of a condition box-car time course with a standard hemodynamic response function built into the Brain Voyager software.

### DTI Analyses

Tensors were reconstructed and tracked from 20-direction diffusion MR images using Diffusion Toolkit Software Version 6.2 and visualized using TrackVis Software Version 5.2 (Wang et al., 2007). The angle threshold was set to 30°. Tracts were preprocessed using a spline filter to remove redundant tract points and segments. Tensors were tracked interactively using regions of interest in the cerebral peduncles and internal capsule to visualize motor fiber tracts as well as sensory pathways in close proximity to the corticospinal tract, such as the corticostriatal and corticothalamic tracts. Diffusion weighted image maps were used as an underlay for anatomical visualization.

### fMRI Results

**Figure 1:**
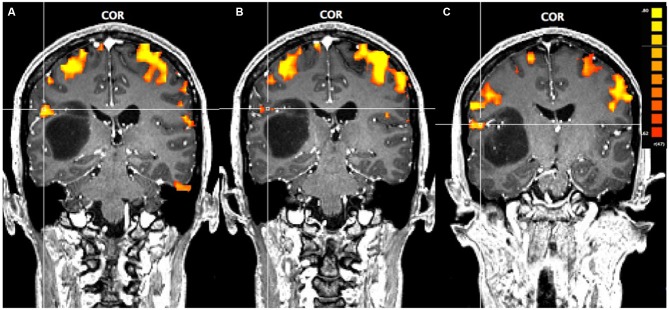
**Pre-operative functional magnetic resonance imaging (fMRI) for the three motor-sensory tasks.** The position of the cross-hairs illustrates the activation of interest nearest to the astrocytoma in each task. **(A)** Activation in the arms rubbing on scanner task. **(B)** Activation in the hands rubbing on scanner task. **(C)** Activation in the lip-licking task.

**Figure 2:**
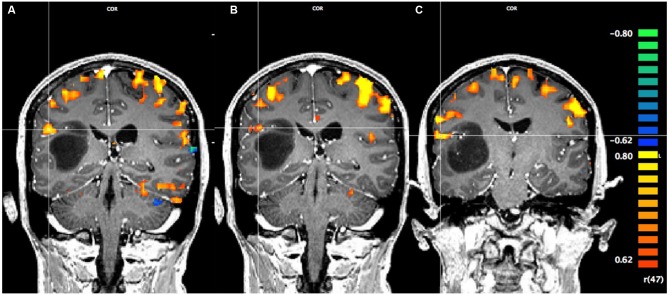
**Pre-operative Talaraich fMRI of the motor and sensory activation, whereby the position of the cross-hairs illustrates the activation of interest nearest to the astrocytoma in each task and the clusters described by the coordinates. (A)** Activation in the arms rubbing on scanner task (coordinates: *x* = 51.7, *y* = −28.8, *z* = 23.1; cluster size: 411 voxels). **(B)** Activation in the hands rubbing on scanner task (coordinates: *x* = 48.6, *y* = −29.2, *z* = 23.6; cluster size: 53 voxels). **(C)** Activation in the lip-licking task (coordinates: *x* = 60.5, *y* = −13.5, *z* = 16.9; cluster size: 185 voxels).

In Figure 1, using a linear correlation threshold of *r* = 0.62 (the observed minimal threshold for eliminating any artifactual activation), we see clear activation in the motor and sensory cortices. This threshold was chosen based on previously published work, where *r* > 0.62 provides an even more conservative threshold of activation than previous studies (e.g., *r* > 0.60; Borowsky et al., 2007; Esopenko et al., 2008; Cummine et al., 2010). Of particular interest, across all three sensorimotor tasks there was activation just superior and lateral to the tumor (which was largely absent in the contra-lateral hemisphere; all *p*’s < 0.0001), resulting in asymmetric activation, possibly related to functional reorganization of the sensorimotor cortex (see Figure 1). As the somatosensory and motor systems are known to have highly consistent and well-mapped somatotopic organization (Penfield and Rasmussen, 1950), and activation in the motor and somatosensory cortices has been shown to have very high concordance with direct cortical stimulation (Kapsalakis et al., 2012), the consistency of this activation throughout the three tasks employed in this experiment provides evidence that this area may have relevant functional implications. A Talaraich transformation was also performed in order to provide standardized coordinates of the activation of interest (Talairach and Tournoux, 1988; see Figure 2). Coordinates and cluster sizes for the arm rubbing, hand rubbing, and lip-licking tasks are *x* = 51.7, *y* = −28.8, *z* = 23.1 and 411 voxels, *x* = 48.6, *y* = −29.2, *z* = 23.6 and 53 voxels, and *x* = 60.5, *y* = −13.5, *z* = 16.9 and 185 voxels, respectively. In addition, language processing is lateralized to the left hemisphere, well removed from the sight of resection.

### DTI Results

**Figure 3:**
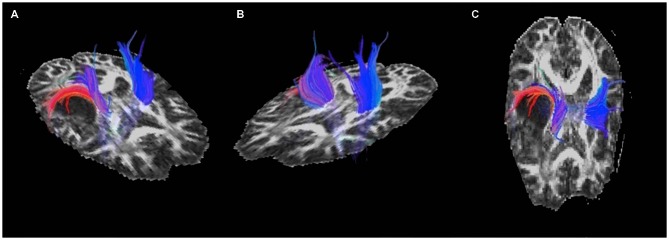
**Pre-operative diffusion tensor imaging (DTI) of the motor and sensory tracts showing (A) and (C) displacement of the corona radiata around the posterior-superior aspect of the astrocytoma and (B) the unaffected tracts in the left hemisphere**.

**Figure 4:**
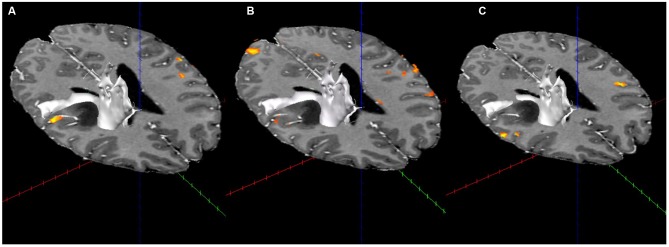
**Combined fMRI and DTI (in white; using Brain Voyager QX Software) for the three fMRI tasks. (A)** Activation with tractography in the arms rubbing on scanner task. **(B)** Activation with tractography in the hands rubbing on scanner task. **(C)** Activation with tractography in the lip-licking task.

Displacement of the corona radiata was found on the right, with many fibers coursing around the superior dorsal surface of the astrocytoma, extending in the direction of the asymmetric activation just superior and lateral to the tumor found using fMRI (see Figure 3), further suggesting cortical redistribution. To further corroborate these findings, combined fMRI/DTI maps were created using Brain Voyager QX Version 2.6.1 software, whereby tracts were both interactively tracked by hand, as well as through a region of interest in the right cerebral peduncle (Figure 4). Tracts were then converted to a mesh for better visualization and overlaid on T1-weighted anatomical images with fMRI activation also presented, whereby the DTI appears to converge upon the location of the activation of interest in each of the three motor tasks.

### Surgical Outcomes

At 6-month follow-up, the patient was left with very mild left weakness and sensory loss due to the surgical procedure. Her motor exam was normal apart from mild generalized weakness, with only mild pronator drift and mild sensory neglect on the left. She also has mild dysmetria on the left and her gait is wide based and shuffling. As well, the patient has had no additional seizures or language deficits.

## Discussion

### Pre-surgical Planning of the Trajectory of Surgical Resection

Based on the fMRI activation that was consistent across all three tasks and the DTI results, a transcortical (rather than transsylvian) superior temporal gyrus surgical approach was chosen. This allowed for avoidance of the tracts identified by DTI that were displaced by the astrocytoma as well as the fMRI activation of interest, in order to minimize potential surgical risks associated with disturbances of this reorganized cortex.

### Surgical Procedure

**Figure 5:**
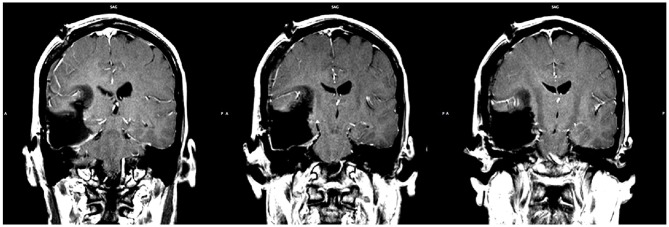
**Post-operative MRI showing the extent of resection.** Gross total resection was achieved (slice indices are approximately *y* = −28, *y* = −29, *y* = −30, from left to right respectively).

A right frontotemporal craniotomy was completed using frameless stereotactic navigation and multimodality neurophysiologic monitoring including electroencephalography, motor evoked potentials, and somatosensory evoked potentials. Based on the strong language lateralization to the left hemisphere exposed via fMRI, awake mapping for speech was deemed unnecessary and was not performed. A gross total resection of the astrocytoma was obtained (Figure 5).

Currently, the primary surgical trajectory for insular gliomas is via the transsylvian fissure (Lang et al., 2001; Signorelli et al., 2010). The use of pre-surgical DTI with a transsylvian approach has been validated through work performed by Wang et al. (2012) in the resection of insular tumors. Their results showed that DTI was useful in identifying areas to be resected as well as predicting clinical function, however fMRI was not employed in this study. In contrast, recent research with cadavers has investigated the optimal trajectory for targeting insular cortex, whereby their results indicate that a transcortical approach through the superior temporal gyrus provides greater insular exposure and surgical freedom compared to the transsylvian approach (Benet et al., 2016). While the utility of fMRI and DTI have been shown *in vivo* using a transsylvian approach (Wang et al., 2012), this case provides evidence that these tools are also valuable in informing a transcortical approach, maximizing extent of resection, and minimizing adverse surgical outcomes related to changes due to sensorimotor plasticity.

The somatotopic organization of the motor and somatosensory cortices and their plasticity has been extensively studied (Buonomano and Merzenich, 1998), mapped, and validated in normal populations, however deviation from this standard somatotopic organization is apparent not only in normal populations (Servos et al., 1999) but also in clinical populations (Schieber, 2001). Of particular importance to this study, lesions have been shown to displace the primary sensorimotor cortices, the white matter tracts associated with these areas, or both. For example, with deep subcortical tumors (e.g., insular tumors), the position of the primary motor areas may be preserved, whereas the corticospinal tract may be displaced, thus disallowing primary motor area activation to be used as a landmark for the corticospinal tract. As such, understanding changes in plasticity at not only the cortical level, but also the subcortical level may provide valuable insight into the potential limits of brain plasticity in both normal and clinical populations.

Further, the tasks developed in this case study may have important implications for reducing fMRI scanning times of the sensorimotor cortex for both normal and clinical populations. We developed a novel set of three experimental tasks to optimally and simultaneously activate the sensory and motor regions proximal to the tumor, specifically the arms (by rubbing arms against scanner wall), hands (by rubbing hands against the scanner), and lips (by licking the lips). Although this was a novel set of experimental tasks, they were very effective at activating the targeted regions of sensorimotor cortex, and we will employ them in future tumor cases where these regions are proximal to a tumor site. Further, although there are limitations whenever using a novel set of tasks, the present results demonstrate their potential utility, and we hope that through continued validation they may become a valuable component of sensorimotor task batteries, supplementing and/or possibly replacing other popular motor and sensory tasks. As such, a basic neuroscience experiment on healthy controls that explores the consistency of activation elicited by these tasks is also warranted. Furthermore, such tasks may also be useful for the developing area of cognitive embodiment research, where there has been considerable interest in examining the embodiment of semantic knowledge near the sensorimotor cortex using functional imaging (Kiefer and Pulvermüller, 2012; Esopenko et al., 2012; Hauk and Tschentscher, 2013). Thus, the ability to examine both sensory and motor activation using a single task is valuable not only for pre-surgical mapping, but also for designing behavioral paradigms for fMRI that maximize sensorimotor activation while minimizing acquisition time.

Importantly, integration of fMRI and DTI measures provides a much more comprehensive picture of function and connectivity than either measure would without the other, especially in tumor cases such as the present one where both functional regions and connections have been disturbed. The development of novel experimental paradigms based on sensorimotor neuroscience, for the purpose of optimizing functional brain activation in a patient, reflect a clear example of “bench-to-bedside” translational research. As we further explore these new tasks in basic research, there is also a reciprocal translation in the other direction, from “bedside-to-bench”.

## Concluding Remarks

In conclusion, fMRI and DTI are being recognized as reliable, non-invasive tools for mapping eloquent cortex in order to improve surgical outcomes (Dimou et al., 2013). High-grade insular astrocytomas pose a special risk based on their close proximity to the corticospinal tracts and increased risk of motor and sensory deficits post-resection (Sanai et al., 2010). This case study demonstrates the value of comprehensive pre-surgical planning of relevant functional cortex (using individually optimized sensorimotor tasks), as well as neural connectivity, in order to minimize surgical risks when using a transcortical approach. As well, this work informs the potential importance of investigating possible neural re-organization that is not visible on standard anatomical scans by integrating both fMRI and DTI, to which damage may result in motor and sensory impairments. It would also be interesting for future research to examine similar anatomical and functional reorganization both before and after surgery and how this may differ as a function of surgical approach. In this case study, pre-surgical planning provided the surgeon with valuable anatomical and functional information in order to optimize the surgical approach through the superior temporal gyrus and maximize the extent of resection, while minimizing adverse side effects. Thus, these techniques can be individualized to inform optimal resection trajectory in order to preserve important functional and structural areas on a case-by-case basis.

## Author Contributions

CLE made substantial contributions to the conception of the work and performed the DTI analyses, created the DTI figures, and drafted the final manuscript for publication. MJSM made substantial contributions to the conception of the work, performed the fMRI analyses and created the fMRI figures. DRF performed the neurosurgery. TE was the neuroradiologist for this case. RWB, LAG, EJL made substantial contributions to the conception of the work, and RWB served as supervisor for CLE, LAG, and EJL. All authors provided critical revisions and approved the final version of the manuscript for publication.

## Conflict of Interest Statement

The authors declare that the research was conducted in the absence of any commercial or financial relationships that could be construed as a potential conflict of interest.

## Footnotes

^1^www.brainvoyager.com
